# Nurturing diversity and inclusion in AI in Biomedicine through a virtual summer program for high school students

**DOI:** 10.1371/journal.pcbi.1009719

**Published:** 2022-01-31

**Authors:** Tomiko Oskotsky, Ruchika Bajaj, Jillian Burchard, Taylor Cavazos, Ina Chen, William T. Connell, Stephanie Eaneff, Tianna Grant, Ishan Kanungo, Karla Lindquist, Douglas Myers-Turnbull, Zun Zar Chi Naing, Alice Tang, Bianca Vora, Jon Wang, Isha Karim, Claire Swadling, Janice Yang, AI4ALL Student Cohort 2020, Bill Lindstaedt, Marina Sirota

**Affiliations:** 1 Department of Pediatrics, UCSF, San Francisco, California, United States of America; 2 Bakar Computational Health Sciences Institute, UCSF, San Francisco, California, United States of America; 3 Department of Bioengineering and Therapeutic Sciences, UCSF, San Francisco, California, United States of America; 4 Cognitive Science and Computer Science Programs, UCLA, Los Angeles, California, United States of America; 5 Program in Biological and Medical Informatics, UCSF, San Francisco, California, United States of America; 6 Department of Pharmaceutical Chemistry, UCSF, San Francisco, California, United States of America; 7 Institute for Neurodegenerative Diseases, UCSF, San Francisco, California, United States of America; 8 Berkeley Institute for Data Science, UC Berkeley, Berkeley, California, United States of America; 9 School of Medicine, UCSF, San Francisco, California, United States of America; 10 Department of Epidemiology & Biostatistics, UCSF, San Francisco, California, United States of America; 11 Quantitative Biosciences Consortium, UCSF, San Francisco, California, United States of America; 12 QBI COVID-19 Research Group (QCRG), San Francisco, California, United States of America; 13 Quantitative Biosciences Institute (QBI), UCSF, San Francisco, California, United States of America; 14 J. David Gladstone Institutes, San Francisco, California, United States of America; 15 Department of Cellular and Molecular Pharmacology, UCSF, San Francisco, California, United States of America; 16 Department of Bioengineering, UC Berkeley, Berkeley, California, United States of America; 17 Saint Francis High School, Mountain View, California, United States of America; 18 AI4ALL, Oakland, California, United States of America; 19 Canton High School, Canton, Michigan, United States of America; 20 Department of Computer Science, Stanford University, Stanford, California, United States of America; 21 Office of Career and Professional Development, UCSF, San Francisco, California, United States of America; 22 Office for Postdoctoral Scholars, UCSF, San Francisco, California, United States of America; McGill University, CANADA

## Abstract

Artificial Intelligence (AI) has the power to improve our lives through a wide variety of applications, many of which fall into the healthcare space; however, a lack of diversity is contributing to limitations in how broadly AI can help people. The UCSF AI4ALL program was established in 2019 to address this issue by targeting high school students from underrepresented backgrounds in AI, giving them a chance to learn about AI with a focus on biomedicine, and promoting diversity and inclusion. In 2020, the UCSF AI4ALL three-week program was held entirely online due to the COVID-19 pandemic. Thus, students participated virtually to gain experience with AI, interact with diverse role models in AI, and learn about advancing health through AI. Specifically, they attended lectures in coding and AI, received an in-depth research experience through hands-on projects exploring COVID-19, and engaged in mentoring and personal development sessions with faculty, researchers, industry professionals, and undergraduate and graduate students, many of whom were women and from underrepresented racial and ethnic backgrounds. At the conclusion of the program, the students presented the results of their research projects at the final symposium. Comparison of pre- and post-program survey responses from students demonstrated that after the program, significantly more students were familiar with how to work with data and to evaluate and apply machine learning algorithms. There were also nominally significant increases in the students’ knowing people in AI from historically underrepresented groups, feeling confident in discussing AI, and being aware of careers in AI. We found that we were able to engage young students in AI via our online training program and nurture greater diversity in AI. This work can guide AI training programs aspiring to engage and educate students entirely online, and motivate people in AI to strive towards increasing diversity and inclusion in this field.

## Author summary

Artificial Intelligence (AI) has the power to improve our lives through a wide variety of applications, many of which fall into the healthcare space; however, a lack of diversity is limiting how broadly AI can impact people. The UCSF AI4ALL program was established to give high school students from underrepresented backgrounds in AI a chance to learn about AI in biomedicine, partake in hands-on application of AI to a research project, and interact with diverse AI role models, including faculty, researchers, and industry professionals. In 2020, the program was changed from an in-person to an online format due to the COVID-19 pandemic. We found that we were able to engage young students in AI via our online training program, deliver an effective curriculum, and nurture greater inclusion in AI. This work can guide AI training programs aspiring to engage and educate students entirely online, and motivate people in AI to strive towards increasing diversity and inclusion in this field.

## 1. Introduction

Artificial Intelligence (AI) has the power to improve our lives through a wide variety of applications. Just a few examples of how AI is being used to enrich our lives include search engines, autonomous vehicles, and facial-recognition, route-planning, and ride-hailing programs [1,2]. The applications of AI to the biomedical, translational, and clinical realms are diverse ranging from discovering biomarkers and repurposing therapeutics, to improving disease diagnosis and automating surgery [2]. Moreover, AI can help realize the promise of personalized medicine, a healthcare approach that aims to tailor medical decisions and treatments to individuals based on their intrinsic (e.g., genomic, age, sex) and extrinsic (e.g., diet, environmental exposures) factors [3].

Yet a lack of diversity can adversely affect how broadly AI will help people [4]. For instance, if machine learning (ML) algorithms to diagnose skin cancer lesions were trained on data that largely represent fair-skinned populations, then the algorithms, no matter how advanced, would not perform as well on images of lesions in skin of darker color [5]. We need diversity not just in the data we use in AI but also in the people working and leading in the field of AI. Currently, AI professors are mostly male (>80%), and among AI researchers, only 15% at Facebook and 10% at Google are female [6].

The UCSF AI4ALL program, established in 2019, strives to promote greater diversity and inclusion in the field of AI in biomedicine, and inspire tomorrow’s leaders to think about and know AI and to use AI ethically. UCSF AI4ALL prioritizes recruiting high school students from backgrounds underrepresented in AI, including females. Through this tuition-free three-week summer training program, students gain experience with AI with a focus on applications to biomedicine, interact and work with a diverse set of role models in AI, and learn about how AI can advance health. They receive broad exposure to AI topics through faculty lectures, and gain in-depth research experience through hands-on projects. Mentoring and career/personal development sessions with faculty, researchers, industry professionals and undergraduate and graduate students further enable personal growth and an opportunity to explore career interests at the intersection of Computer Science and Biomedicine. Due to the COVID-19 pandemic, the UCSF AI4ALL program held in 2020 shifted from an in-person, commuter program to a synchronized, online one with all the student research projects focusing on leveraging AI to advance our knowledge and understanding of COVID-19. Here, we provide an overview of the 2020 UCSF AI4ALL virtual summer program, share details about the research projects our students engaged in, and discuss the results of our program.

## 2. Methods

**Ethics statement.** Approval for this study was granted by the UCSF IRB (# 21–34523). Formal consent was not obtained as (a) the data was collected anonymously, and (b) the study is considered secondary research for which consent is not required.

**Cohort selection.** We reviewed all complete applications that were submitted to our program during the application period in March (48 applications in 2019, 89 applications in 2020, 209 applications in 2021), and assessed each candidate holistically prior to offering acceptances into our program. Application reviewers assessed students’ transcripts, letters of recommendation, and short essay responses to questions asking applicants “Why do you want to participate in UCSF AI4ALL Program?”, “What role do you think technology can play in biomedicine and healthcare?”, “Tell us about a time when you faced a challenge and describe how you overcame it.” Our selection prioritized applications with essay responses that expressed a strong level of enthusiasm. Our selection especially prioritized applications from students who noted that they were facing / overcoming challenges (e.g., from low-income background, will be future first-generation college student), appeared motivated to learn (taught themselves how to do something challenging for them), and/or from underrepresented background in AI (e.g., Indigenous Peoples, Black, Hispanic or Latinx, Pacific Islander, and Southeast Asian, and girls).

**Instructional details.** The program itself is a three-week program, which has been held virtually since 2020. The learning objectives for the students were to (a) understand concepts in AI, (b) develop foundational skills in basic programming and machine learning, and (c) appreciate how these skills can be applied to research. Furthermore, an important goal of the program for the students was for them to build connections with their peers and mentors.

The first week focused on teaching the high school participants basic programming in Python, and about ethics in AI, data and bias, and introductory topics in machine learning. The second and third weeks focused on research projects, which in 2020 were all leveraging AI to COVID-19. Google CoLab notebooks were used as a simple means to share code with students and run Python code. Each morning began with time for students to ask questions as well as participate in icebreaker activities. The program had guest lectures by diverse faculty from UCSF as well as panels composed of UCSF AI4ALL student alumni, undergraduate and graduate students, and industry professionals who talked about their lives, their work, and applications of AI in biomedicine. Each week, our Alumni TAs led a Community Building Session engaging the class of students in fun, bonding exercises. We also held a personal growth session to develop the students’ communication skills. The end of the program symposium included student presentations on their research projects as well as a Keynote talk on AI in Biomedicine. An account of each day of the 2020 program was chronicled on our blog, https://medium.com/ucsfai4all.

Program materials, including teaching and research project resources, are available:

- 2020 Program Schedule (S1 Text)
- 2020 Lecture Videos Links (S2 Text)
- Icebreaker and Community Building Ideas (S3 Text)
- Personal Growth: Communication Workshop Slides (S4 Text)
- 2020 Research Projects Descriptions (S5 Text)

### 2.1 Community building, guest faculty lectures, panels, and personal growth

The first half hour of each morning was dedicated to answering any questions the students had as well as for our icebreaker activities in which all students participated. These icebreaker activities included asking each student to share a different use for an ordinary item (e.g., a leaf blower) or answer questions like, “If you could only have three foods with you on a deserted island, what would those be?”. These sessions were followed each morning with a guest faculty lecture or panel session. The guest lectures were given by diverse faculty from UCSF who talked about how they became interested in their work and what school was like for them, and also focused on application of AI in biomedicine and covering a wide range of topics from clinical data analysis, to diagnostic and therapeutic strategies leveraging molecular measurements. There were also several panels, with each panel composed of UCSF AI4ALL student alumni, undergraduate students, graduate students, or professionals from private companies with backgrounds in AI within biomedicine and other disciplines. Our guest faculty speakers also touched upon issues such as ethics, bias, and privacy during their talks. Our panelists, many of whom were women and people from diverse racial and ethnic backgrounds, spoke with our students and shared insights about their work and their journeys.

One day each week in the afternoon, our Alumni TAs led a 1.5-hour Community Building Session engaging the class of students in various fun, bonding exercises, including Kahoot, Scribbl, and Scattergories. Depending on the activity, the students interacted with each other in one large group or in smaller groups of 5 to 10 students in break-out rooms. Icebreakers and Community Building Activity Ideas are available (S3 Text).

We also held a 1-hour personal growth workshop to develop the students’ communications skills. This session focused on helping students construct an “Elevator Pitch” about themselves so they could become more comfortable and skilled at briefly talking about themselves and their accomplishments. The slides from this workshop are available (S4 Text).

### 2.2 1^st^ week: Lessons in python and machine learning

In the first week of the program, students spent the afternoons learning about machine learning concepts and programming in Python. We had seven to nine UCSF graduate student instructors and teaching assistants (TA) to help with teaching during the first week. Depending on the number of sessions taught, graduate students spent 1 to 4 hours on teaching this week. iPython notebooks with the in-class exercises were shared the evening before the class, to give students an opportunity to practice on their own before the solutions were reviewed in class.

#### 2.2.1 Python workshops

Students covered the basics of programming, data management, and data visualization in the first two days to prepare to code in Python language and work with data within a Google CoLab environment in preparation for their projects. Topics covered include programming basics (data types, logic, loops, functions), data structures, common Python packages, plotting with *matplotlib*, and using *sklearn*. During the lesson, students were placed in breakout rooms with teaching assistants, with each teaching assistant working with 5 to 8 students in a breakout room, to review coding exercises and practice programming activities together.

#### 2.2.2 Lessons on machine learning

Topics covered in ML include Data and Bias, Clustering, Classification, Naive Bayes, Regression, and Neural Networks. To facilitate remote instruction, we employed an inverted classroom paradigm, in which the instructors produced a 15–20-minute lecture video to be watched before each classroom session. The general structure of the 45-minute-long live sessions includes 15 minutes of topic review first, then the rest of the session covering either conceptual activities or reviewing and practicing ML exercises on CoLab notebooks. For activities, students were placed into breakout rooms with a teaching assistant. Since students come from various backgrounds of ML and programming familiarity, collaboration within the breakout rooms was encouraged.

The instruction was carried out in an inverted classroom setting where the participants could ask the instructors and TAs questions after having watched the lectures. These lecture videos are available (S2 Text)

### 2.3 2nd and 3rd weeks: Research projects and presentations

#### 2.3.1 Applying AI to COVID-19

The students were assigned to one of five groups based on their preference, each working on a research project that applied AI to the characterization, classification, or prediction of COVID-19 leveraging different types of biomedical data—gene expression, proteomics, imaging and clinical. Each project team was led by a UCSF graduate student, medical student, and/or postdoctoral scholar and co-led by an alumni TA. These research teaching assistants spent approximately 5 to 20 hours per week on teaching during this two-week period.

In one of the projects, students used publicly available daily time series data describing confirmed COVID-19 infections and deaths per country and states across the world (over 266 regions) aggregated from the Johns Hopkins Center for System Sciences downloaded on July 1, 2020 (https://github.com/CSSEGISandData) [7] to develop an algorithm that can predict the number of cases in a given country. In another project, students learned to implement supervised learning techniques to predict host protein interactors, given primary amino acid sequences of various viral proteins, and to test if proteins of similar sequences would interact with the same host proteins. Students learned to implement machine learning models that can classify COVID-19 cases in chest x-ray images. Students also leveraged metagenomic next generation RNA sequencing (mNGS) to study patient subgroups of COVID-19 through exploratory and unsupervised analyses. Finally, using real world data from (https://www.kaggle.com/einsteindata4u/covid19) [8], students learned how to apply AI to (A) predict whether a patient is COVID positive or negative and (B) predict the severity of the COVID infection (i.e. admission into the general ward, semi-intensive care unit, or intensive care unit). Descriptions of research projects and associated data and code are shared (S5 Text).

During the Final Symposium at the end of the program, students shared broadly with an audience of invited guests the findings from their group’s research project. Each presentation was approximately 15 to 20 minutes in length, with time for questions, and each student presented a portion of their group’s work.

**Assessment methods.** Students were asked to complete a survey at the beginning (Pre-) and at the conclusion (Post-) of our program. Mann Whitney U (MWU) test with continuity correction was used to compare Pre- to Post- survey responses (since surveys were anonymous, we could not compare these using tests designed for paired data), and Kruskal-Wallis (KW) test was used to compare 2019 to 2020 and 2021 Pre- and Post- survey responses. Bonferroni corrections were employed, and a significance threshold of 0.05 was applied to the results.

## 3. Results

### 3.1 Students

Of the 89 high school students who submitted applications to our 2020 program and the 38 applicants we accepted into the program, 29 enrolled in and completed the program.

All 29 students were females who were rising sophomores (21%), juniors (45%) or seniors (34%) in high school. Most of the students were from California (79%), although several were from other states. The racial backgrounds of the students included Asian inclusive of those from the Indian subcontinent and Philippines (79%), Native Hawaiian or Other Pacific Islander/Original Peoples (3%), and Hispanic or Latino (7%), and 14% declined to state. Twenty-one percent will be first generation college students. (**Table 1**). In 2019, 18 of the 48 students who submitted applications to this in-person program were accepted into and completed the program. In 2021, 27 of the 209 students who submitted applications to our virtual program were accepted into and completed the program. Demographic characteristics of students in the 2019 and 2021 programs are summarized in **S1 and S2 Tables**.

**Table 1:** Demographic characteristics of accepted students in the 2020 UCSF AI4ALL Summer program.

Characteristic	# students (total accepted: 29)	% students
**Gender**		
She/Her	29	100%
He/Him	0	0%
They/Them	0	0%
**Race** *more than one category may be checked		
Asian (including Indian subcontinent and Philippines)	23	79.3%
Black or African American	0	0%
Native Hawaiian or Other Pacific Islander (Original Peoples)	1	3.4%
Hispanic or Latino (including Spain)	2	6.9%
White (including Middle Eastern)	0	0%
Decline To State	4	13.8%
**Grade Level Next Year**		
Senior / 12th grade student	10	34.4%
Junior / 11th grade student	13	44.8%
Sophomore / 10th grade student	6	20.7%
Freshman / 9th grade student	0	0%
**School**		
Public	23	79.3%
Private	6	20.7%
Charter	0	0
**Qualify for Free Lunch at School**		
Yes	4	13.8%
No	25	86.2%
**1st Gen College Student**		
Yes	6	20.7%
No	23	79.3%
**Home State**		
California	23	79.3%
Other	6	20.7%

### 3.2 Participation and presentations

All 29 students who attended our virtual AI program completed the three-week training. They participated in a variety of sessions, including the Python workshops, machine learning lessons, guest faculty lectures, a personal growth session, community building events, and panel discussions. Through these sessions, students interacted with AI role models, including faculty, high school students, undergraduate and graduate students, and industry professionals, many of whom were women and people from diverse racial and ethnic backgrounds. Each student also engaged in one of our five hands-on small-group research projects that applied AI to COVID-19.

On the last day of the program, the students shared findings from their group’s research project during our Final Symposium. Each student presented a portion of their group’s work, and each presentation was approximately 15–20 minutes including time for them to answer questions from the audience. The event was attended virtually by over 100 people, including faculty, graduate students and postdoctoral scholars from UCSF and other academic institutions, program participants and their invited family members. A videorecording of the 2020 Final Symposium, including our Keynote Speaker’s talk and the students’ presentations is available, https://youtu.be/uImjiHl7MDw. Also available are video recordings of the Final Symposium for the programs held in 2019, https://lecture.ucsf.edu/ets/Play/ae367585491f4ca88da88792e96536f31d, and in 2021, https://youtu.be/jccq2gIJ06I. These videos capture not only the work that our students were able to complete within two weeks on their research projects—from data wrangling to the application of machine learning models to the interpretation of their results–but also their ability to convey their work to a broad audience.

Our blog, https://medium.com/ucsfai4all, chronicles each day of the three-week program.

### 3.3 Pre- and post- surveys

**Fig 1:**
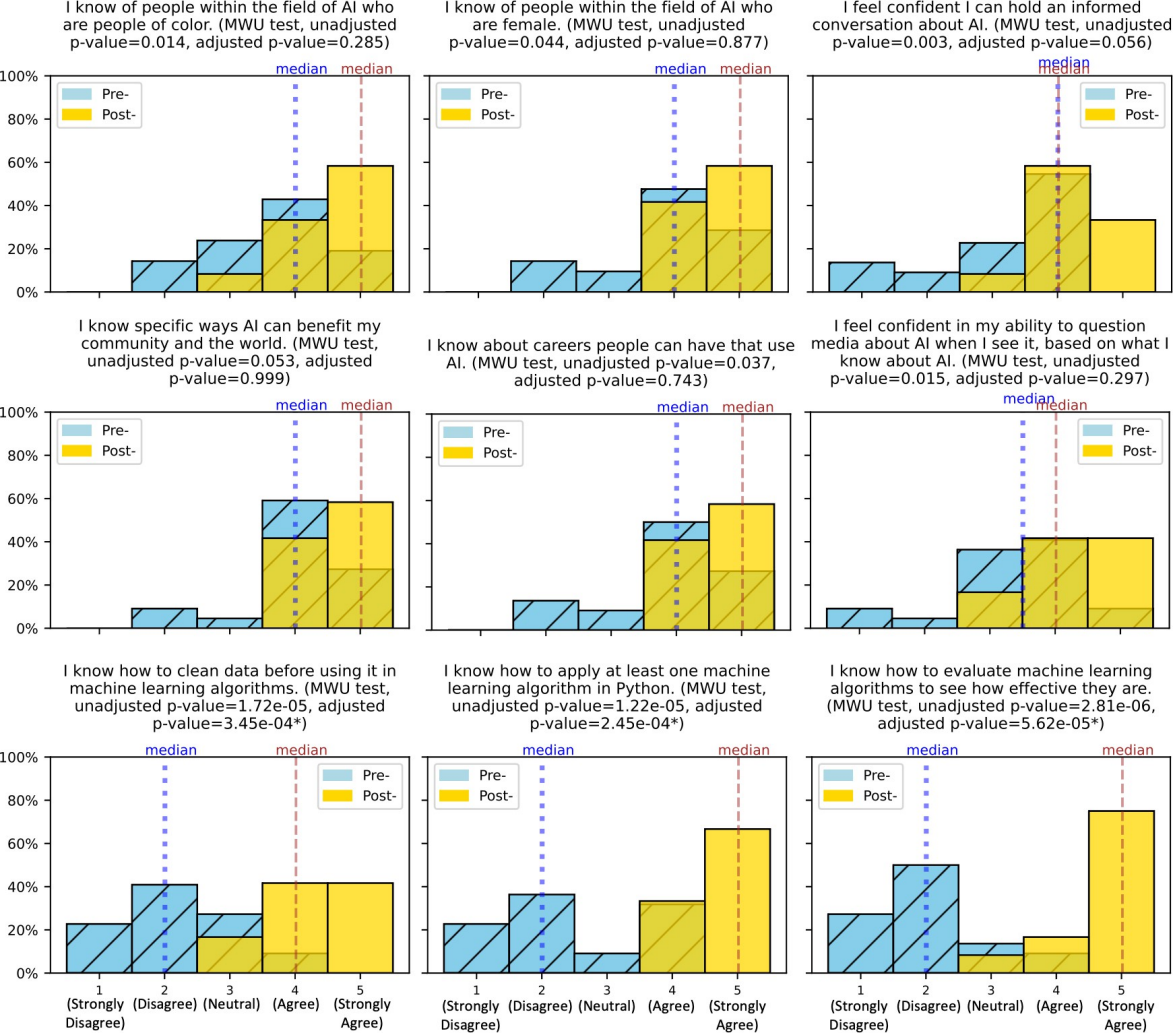
Pre-post survey results. Histograms of students’ responses to pre-program and post-program survey questions, with median values. Mann Whitney U tests were performed to compare pre- and post-survey responses, with adjusted p-values below significance threshold of 0.05 given an asterisk (*).

The survey response rates in 2019 were 100% (18/18) for Pre- and 55.56% (10/18) for Post-surveys, in 2020 were 75.86% (22/29) for Pre- and 41.37% (12/29) for Post-surveys, and in 2021 was 77.78% (21/27) for Pre- and 92.59% (25/27) for Post-surveys. Analysis of the survey data revealed significant shifts in some of the students’ responses from the Pre- to Post- survey. Specifically, at the end of the program, there were significantly more students who reported that they know how to clean data before using it in machine learning algorithms (MWU test, adjusted p-value <0.001), and know how to evaluate and apply machine learning algorithms (MWU test, adjusted p-values <0.001) (**Fig 1**). More students also reported knowing people in AI who are people of color (MWU test, unadjusted p-value = 0.014, adjusted p-value = 0.285) and women (MWU test, unadjusted p-value = 0.044, adjusted p-value = 0.877), feeling confident in questioning the media about AI (MWU test, unadjusted p-value = 0.015, adjusted p-value = 0.297), and knowing about careers that use AI (MWU test, unadjusted p-value = 0.037, adjusted p-value = 0.743); however, these increases were only of nominal significance. Survey responses of students in the 2020 and 2021 virtual programs and those of students in the 2019 commuter program for questions asked across years were found not to differ significantly (KW test, unadjusted and adjusted p-values > 0.05) (**S3 Table**). Students of the 2020 virtual program were also as likely to recommend the AI4ALL program to peers than the students who attended the 2019 in-person program (MWU test, unadjusted p-value = 0.044, adjusted p-value = 0.872) (**S1 Fig**).

Moreover, the graduate students, post-doctoral scholar and alumni TA’s who worked with our students on their research projects shared their observations that the students:

○ Grew in their abilities to think critically, form hypotheses, and design executable experiments
○ Learned how to develop a variety of different ML models
○ Fortified their collaborative skills and technical proficiency in Python
○ Found exploratory data analysis to be valuable as it challenged the participants to view scientific inquiry in an open-ended way that deviated from traditional classroom experiences
○ Learned how to analyze the results and how they relate to the original research questions
○ Experienced the immediate relevancy of AI approaches to current problems in the COVID-19 pandemic
○ Demonstrated their understanding and interpretation of not only AI but also the application of AI to medicine, public health, and clinical decision making

## 4. Discussion

Diverse representation is needed not just in the data for Artificial Intelligence (AI), but also in the people working and leading in the field of AI. Since 2019, UCSF AI4ALL has engaged students from backgrounds historically underrepresented in AI in order to promote greater diversity and inclusion in this field. In 2020, through a variety of interactive virtual real-time sessions and experiences held during a three-week period, our program allowed students to interact with a diverse set of role models in AI and learn about how AI can be used to advance health. Furthermore, students gained experience in coding, working with data, and AI by participating in one of our meaningful hands-on research projects that applied AI to understanding, classifying, or predicting COVID-19.

Students’ survey responses demonstrated their feeling significantly more familiar with working with data and evaluating and applying machine learning algorithms at the end of our 2020 virtual program. There was also a nominally significant increase in the students’ knowing people in AI who are from historically underrepresented groups, their confidence in discussing AI, and their awareness of careers in AI. While the format of the 2020 and 2021 programs differed from the 2019 program, with the 2020 and 2021 programs taking place online instead of in-person due to the pandemic, students’ survey responses from these three years were comparable.

Based on the fast-paced nature of our program, we now share information about optional, self-paced online Python training that we advise students to take before participating in our program. We also provide information about online resources so that students can review basic biology concepts (e.g., DNA replication, RNA transcription and translation, central dogma, DNA sequencing). We hope that by sharing these resources, students with less prior experience will be able to participate and flourish in our program.

Despite the success of our virtual training program, there were some limitations to having a program take place entirely online, including the lack of in person interactions and the need for reliable internet connection. Nevertheless, the ability to engage young students in AI and the opportunity to contribute to diverse representation in this field make holding our program in any format worthwhile.

## 5. Conclusion

We have learned that it is possible to deliver virtually an AI in Biomedicine curriculum to diverse, young high school students that provides them with an engaging and impactful experience. Through our virtual program, we were able to connect with students from around the country and involve diverse teaching assistants and faculty from outside the Bay Area and from other institutions. We were also able to give students who are located far from AI training programs a chance to become involved bringing the goal of increasing diversity in AI a little closer to reality.

## Supporting information

S1 Fig Likeliness to recommend program in 2019 vs 2020. Histogram of 2019 and 2020 students’ responses to how likely they were to recommend the AI4ALL summer program to a peer (question not asked in 2021). Mann Whitney U tests were performed to compare responses.
(TIFF)

S1 Table Demographic characteristics of students in the 2019 UCSF AI4ALL Summer program. (XLSX)

S2 Table Demographic characteristics of students in the 2021 UCSF AI4ALL Summer program. (XLSX)

S3 Table Comparison of 2019, 2020, and 2021 survey responses. Medians and unadjusted and Bonferroni-adjusted p-values from Kruskal-Wallis (KW) test for comparison of survey responses of students in the 2020 and 2021 virtual programs and in the 2019 commuter program for questions asked across the years. 1: strongly disagree, 2: disagree, 3: neutral, 4: agree, 5: strongly agree. Bonferroni-adjusted p-values from Kruskall-Wallis (KW) test. na: not asked on survey.
(XLSX)

S1 Text 2020 Program Schedule. (PDF)

S2 Text 2020 Program Lecture Videos. Links to the lecture videos.
(DOCX)

S3 Text Icebreaker and Community Building Ideas. (DOCX)

S4 Text Personal Growth: Communication Workshop Slides. (PDF)

S5 Text 2020 Research Projects. Descriptions of the 5 research projects for the 2020 program with links to code repositories and available data.
(DOCX)
