# Supplementary figures and images for: Nurturing diversity and inclusion in AI in Biomedicine through a virtual summer program for high school students
Source: PLoS Comput Biol. 2022 Jan 31;18(1):e1009719. doi: 10.1371/journal.pcbi.1009719 (PMC8830787; doi:10.1371/journal.pcbi.1009719)

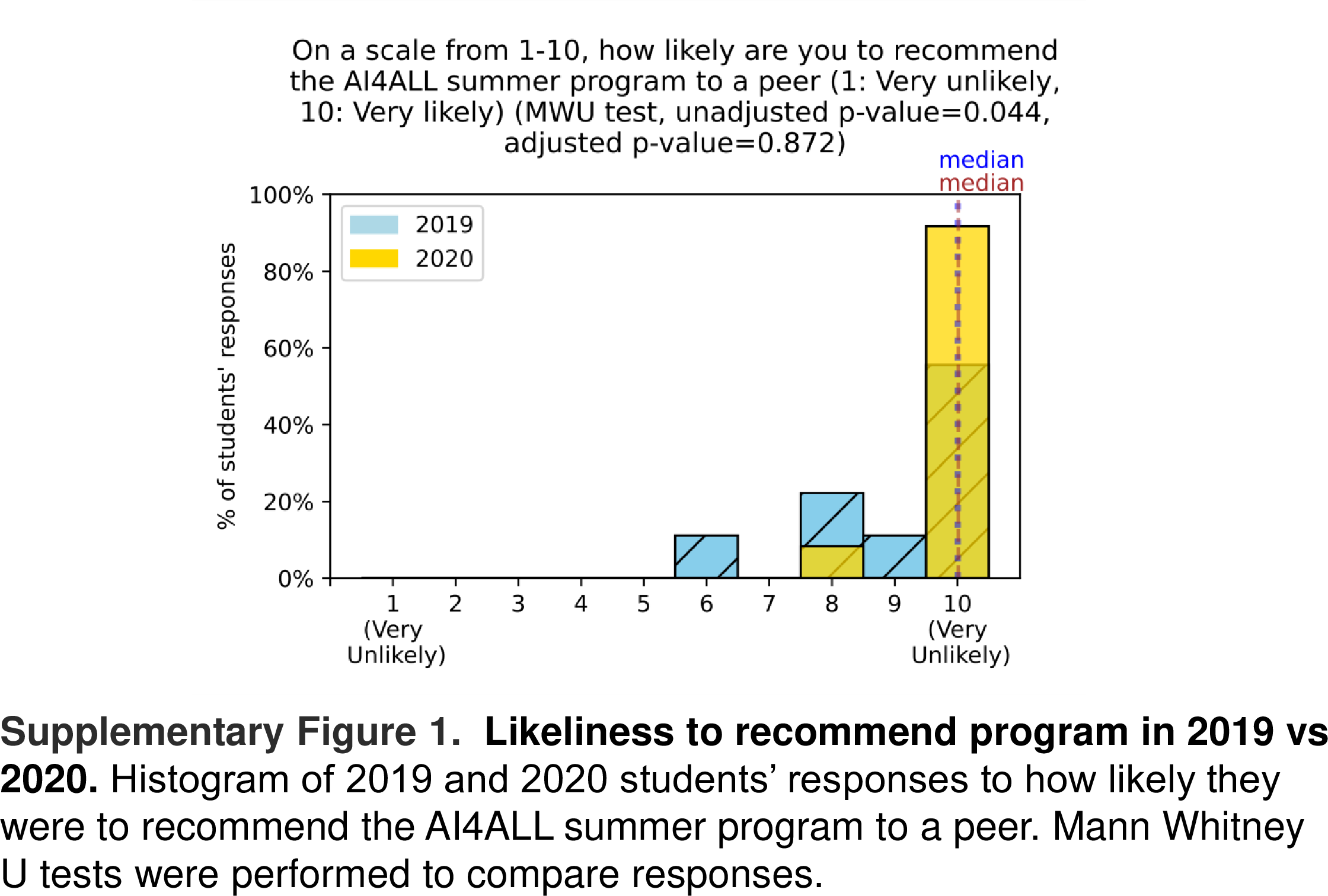

Supplement: S1 Fig — Histogram of 2019 and 2020 students’ responses to how likely they were to recommend the AI4ALL summer program to a peer (question not asked in 2021). Mann Whitney U tests were performed to compare responses. (TIFF) [file pcbi.1009719.s001.tiff]
