# Supplementary material for: Nurturing diversity and inclusion in AI in Biomedicine through a virtual summer program for high school students
Source: PLoS Comput Biol. 2022 Jan 31;18(1):e1009719. doi: 10.1371/journal.pcbi.1009719 (PMC8830787; doi:10.1371/journal.pcbi.1009719)
Supplement: S1 Text — (PDF) [file pcbi.1009719.s005.pdf]

Legend
Food/Break/Non-curricular
Lecture / Core Curriculum Time
Project Time
Guest Speaker / Field Trip / Panel
Personal Growth session

Time	Monday, July 13<br>Day 1	Tuesday, July 14<br>Day 2	Wednesday, July 15<br>Day 3	Thursday, July 16<br>Day 4	Friday, July 17<br>Day 5
10:00 AM	Program Intro, Schedule, and Expectations	Welcome / Break out rooms	Welcome / Break out rooms	Welcome / Break out rooms	Welcome / Break out rooms
10:15 AM	Intro from AI4ALL	Questions from day before	Questions from day before	Questions from day before	Questions from day before
10:30 AM	Guest Speaker (**Marina Sirota**)	Guest Speaker (**Matt Spitzer**)	Guest Speaker (**Atul Butte**)	Guest Speaker (**Sergio Baranzini**)	Guest Speaker (**Sara Murray**)
10:45 AM					
11:00 AM					
11:15 AM					
11:30 AM					
11:45 AM					
12:00 PM					
12:00 PM	Lunch	Lunch	Lunch	Lunch	Lunch
12:15 PM					
12:30 PM					
12:45 PM					
1:00 PM	Lesson 1: Introduction to AI	Lesson 3: Introduction to Machine Learning	Lesson 5: Classification	Lesson 7: Regression	Introduction to Projects
1:15 PM					
1:30 PM					
1:45 PM	Break	Break	Break	Break	
2:00 PM	Lession 2: Data and Bias	Lesson 4: Clustering	Lesson 6: Naive Bayes	Lesson 8: Neural Networks	
2:15 PM					
2:30 PM					
2:45 PM	Break	Break	Break	Break	Break
3:00 PM	Python P1: Variables, Loops, Etc.	Python P3: Jupyter Notebooks & Data Exploration	Python P4, Day1: Data Exploration & Scikit-Learn Basics	Python P4, Day2: Data Exploration & Scikit-Learn Basics	Community Building
3:15 PM					
3:30 PM	Python P2: Functions and Libraries				
3:45 PM					
4:00 PM					
4:15 PM					
4:30 PM					
4:45 PM					
5:00 PM					

Legend
Food/Break/Non-curricular
Lecture / Core Curriculum Time
Project Time
Guest Speaker / Field Trip / Panel
Personal Growth session

Time	Monday, July 20<br>Day 6	Tuesday, July 21<br>Day 7	Wednesday, July 22<br>Day 8	Thursday, July 23<br>Day 9	Friday, July 24<br>Day 10
10:00 AM	Welcome / Break out rooms	Welcome / Break out rooms	Welcome / Break out rooms	Welcome / Break out rooms	Welcome / Break out rooms
10:15 AM	Questions from day before	Questions from day before	Questions from day before	Questions from day before	Questions from day before
10:30 AM	Guest Speaker (**Sourav Bandyopadhyay**)	Undergraduate Student Panel	Graduate Student Panel	Guest Speaker (**Gabi Fragiadakis**)	AI4ALL Alumni Panel
10:45 AM					
11:00 AM					
11:15 AM					
11:30 AM					
11:45 AM					
12:00 PM					
12:00 PM	Lunch	Lunch	Lunch	Lunch	Lunch
12:15 PM					
12:30 PM					
12:45 PM					
1:00 PM	Project Time	Guest Speaker (**Ida Sim**)	Project Time	Project Time	Personal Growth
1:15 PM					
1:30 PM					
1:45 PM					
2:00 PM					
2:15 PM					
2:30 PM		Break			Break
2:45 PM	Break		Break	Break	
3:00 PM	Project Time	Project Time	Community Building	Project Time	Project Time
3:15 PM					
3:30 PM					
3:45 PM					
4:00 PM					
4:15 PM					
4:30 PM					
4:45 PM					
5:00 PM					

Legend
Food/Break/Non-curricular
Lecture / Core Curriculum Time
Project Time
Guest Speaker / Field Trip / Panel
Personal Growth session

Time	Monday, July 27<br>Day 11	Tuesday, July 28<br>Day 12	Wednesday, July 29<br>Day 13	Thursday, July 30<br>Day 14	Friday, July 31<br>Day 15
10:00 AM	Welcome / Break out rooms	Welcome / Break out rooms	Welcome / Break out rooms	Welcome / Break out rooms	Welcome / Break out rooms
10:15 AM	Questions from day before	Questions from day before	Questions from day before	Questions from day before	Questions from day before
10:30 AM	Guest Speaker (**Julian Hong**)	AI4ALL Industry Professional Panel	Bio in AI Industry Professional Panel	Guest Speaker (**Michael Keiser**)	Project Time
10:45 AM					
11:00 AM					
11:15 AM					
11:30 AM					
11:45 AM		Lunch			
12:00 PM					
12:00 PM	Lunch		Lunch	Lunch	Lunch
12:15 PM					
12:30 PM		Project Time			
12:45 PM					
1:00 PM	Project Time		Project Time	Project Time	Symposium Keynote
1:15 PM					
1:30 PM					
1:45 PM					
2:00 PM					Project Presentations
2:15 PM					
2:30 PM					
2:45 PM	Break	Break	Break	Break	
3:00 PM	Project Time	Project Time	Community Building	Project Time	
3:15 PM					
3:30 PM					Where To Go From Here + AI4ALL Alumni Community Talk
3:45 PM					
4:00 PM					
4:15 PM					
4:30 PM					
4:45 PM					
5:00 PM					
