# Supplementary material for: Nurturing diversity and inclusion in AI in Biomedicine through a virtual summer program for high school students
Source: PLoS Comput Biol. 2022 Jan 31;18(1):e1009719. doi: 10.1371/journal.pcbi.1009719 (PMC8830787; doi:10.1371/journal.pcbi.1009719)
Supplement: S2 Text — Links to the lecture videos. (DOCX) [file pcbi.1009719.s006.docx]

**Supplemental Material 1. 2020 Lecture Videos**

| **Video** | **Link** |
| --- | --- |
| Lesson 1:  Intro to AI (10m 00s) | <https://youtu.be/HJ5fsHzTU68> |
| Lesson 2:   Data & Bias (14m 27s) | <https://youtu.be/By4MiU4vIYc> |
| Lesson 3: Intro to Machine Learning (16m 31s) | <https://youtu.be/xvTU9d_bwuk> |
| Lesson 4:  Clustering (16m 41s) | <https://youtu.be/ERYZH7lkL48> |
| Lesson 5:  Classification (18m 59s) | <https://youtu.be/QvgOS896mJI> |
| Lesson 6:  Naive Bayes (16m 31s) | <https://youtu.be/iB4d7PyrDnw> |
| Lesson 7:  Regression (15m 46s) | <https://youtu.be/aqHR49BrdbE> |
