# Supplementary material for: Nurturing diversity and inclusion in AI in Biomedicine through a virtual summer program for high school students
Source: PLoS Comput Biol. 2022 Jan 31;18(1):e1009719. doi: 10.1371/journal.pcbi.1009719 (PMC8830787; doi:10.1371/journal.pcbi.1009719)
Supplement: S3 Text — (DOCX) [file pcbi.1009719.s007.docx]

**Icebreakers and Community Building Ideas**

**Welcome/Breakout Rooms, first 30 minutes of each day**

1. Random question to answer
2. Would you rather?
3. Uses for a common item (pencil)
4. Week 1: Monday:
   1. Our spotlights + explain roles + sneak peak of teams
5. Rest of Week 1 — spotlights
   1. Friday - kudos
   2. Their spotlights (2-3 per day)
   3. Calming coloring (soft music, just talk)

**Community Building Time, 1.5 hour session each week**

1. Week 1:
   1. Put into fake groups based on ‘similarity’ in application
      1. Split students into random groups and tell them it was based on a similarity in their application. Have them discuss to try and find it (tell them later there actually wasn’t :))
   2. Trivia Kahoot ([link](https://create.kahoot.it/search?query=trivia&tags=trivia&filter=filter%3D1)) (20 questions)
   3. Speed Dating (2 people, 3 minutes each, 6-7 rotations, students introduce themselves to each other)
   4. 2 OPTIONS
      1. Zoom charades (1 by 1)
         1. moderator
      2. Just talk
   5. Line-by-line story ([instructions](https://www.fatherly.com/play/this-simple-storytelling-game-flexes-your-kids-creative-muscles/))
2. Week 2:
   1. Personal Trivia Kahoot ([create Kahoot](https://create.kahoot.it/creator))
      1. Give us 3 fun facts about you
   2. Adapted Mafia ([instructions](https://en.wikipedia.org/wiki/Mafia_(party_game)), use private messages)
      1. Breakout rooms, 3 of 10
      2. 2 rounds break out rooms
   3. Zoom Charades
      1. Moderator, popcorn style for who goes next
   4. Speed Dating ([instructions](https://youthgroupgames.com.au/games/speed-dating-with-a-twist/))
      1. 2 people, 4-5 min, 6-7 rounds
   5. Scribbl, <https://skribbl.io>
      1. 6 groups of 5
3. Week 3:
   1. Write a letter to your future self
   2. Scattergories [https://scattergoriesonline.net/](https://scattergoriesonline.net)
   3. Movie Trivia

Other Ideas

- 2 truths and a lie (regular or guess the person?)
- Kudos time (have a google form) — read at end of week
- Related to teams
  - Spirit week/day (wearing team colors)
- Apples to apples/cards against humanity made by us: <https://allbad.cards>
- Some kind of educational game
- Improv
- Pitching for different fun problems
- Extra cool AI stuff: <https://experiments.withgoogle.com/collection/ai>

Random List of Questions

1. If you were a cat that ate a zamboni, what would you tell your mom?
2. Why would you change insurances, as a gorilla?
3. Do you think you smell like formaldehyde? You don’t think so--but consider if it was really true?
4. What is your most embarrassing memory?
5. Have you ever seen your dad comb his hair?
6. Would you embrace a cold bracelet?
7. How would you feel if trees were celebrities?
8. Would you toast a burnt marshmallow?
9. Your mom wants you to go to boarding school. Your sister thinks you should buy a submarine. What do you do?

Superlatives

1. Best person to share a deserted island with
2. Most likely to visit Mars
3. Most likely to start a band
4. Most likely to use a leaf blower as a hairdryer
5. Most likely to meet Tony the orangutan
6. Most likely to catch their marshmallow on fire while roasting it
7. Most likely to secretly be having a party while their camera is off
8. Most likely to remember everyone’s names
9. Most likely to be addicted to Starbucks
10. Most likely to survive the Hunger Games
11. Most likely to live in a submarine
12. Most likely to buy a cat just to pet it
13. Most likely to drink coffee for fun
14. Most likely to publish a manifesto
15. Most likely to run a Kissing Booth
16. Most likely to craft a hourglass out of paper cranes
17. Most likely to buy medicine for a sleeping turtle
18. Most likely to use a paper cup as a plastic cup
19. Most likely to take a llama to a Walmart
20. Most likely to use a donkey for moral support
21. Mostly likely to become a talk show host
22. Most Likely to Cry if You Ask “Are You Gonna Cry?”
23. Most likely to run a marathon
24. Most likely to do jazz hands
25. Most likely to become a Sherlock Holmes
26. Most likely to become a film director
27. Most likely to backpack all over the world
28. Most likely to be on the *Simpsons*
29. Most likely to be part of a real like King Kong situation
30. Most likely to cry at the end of every movie
