# Supplementary material for: Nurturing diversity and inclusion in AI in Biomedicine through a virtual summer program for high school students
Source: PLoS Comput Biol. 2022 Jan 31;18(1):e1009719. doi: 10.1371/journal.pcbi.1009719 (PMC8830787; doi:10.1371/journal.pcbi.1009719)
Supplement: S4 Text — (PDF) [file pcbi.1009719.s008.pdf]

Constructing your Elevator Pitch

talking about yourself, your accomplishments, and your work, quickly

Bill Lindstaedt, MS
Assistant Vice Chancellor
Career Advancement, International and Postdoctoral Services
University of California, San Francisco

Goals:

- Become more **comfortable** at talking about ourselves and our accomplishments, and our work at UCSF

- Become more **skilled** at talking about ourselves and our accomplishments and our work at UCSF

Mission in a minute – UCSF faculty talk about their work at UCSF

Midwifery

https://www.youtube.com/watch?v=WaICdWds4Zk&list=PLVLbDUiGesPyQqgEEsceWk4-FzSmMeI_d&index=3&t=0s

3D models of the beating human heart

https://www.youtube.com/watch?v=rggdf5AMhr8&list=PLVLbDUiGesPyQqgEEsceWk4-FzSmMeI_d&index=13

Palliative care
https://www.youtube.com/watch?v=IPpJXKuLCXw&list=PLVLbDUiGesPyQqgEEsceWk4-FzSmMeI_d&index=20

Common themes of good one-minute videos

- Are easy to understand
- Convey the big picture and why the work is important
- Includes an attention-grabbing statement early
- Provide easy-to-recognize comparisons
- State clear goals for the work
- Convey enthusiasm for the work

What is an Elevator Pitch? For our purposes:

- Any very brief statement about you and/or your work and/or goals and/or needs
- Content, length, style varies based on audience and purpose
- Designed to open further conversation…not to tell everything
- Follows a structure, outline or framework
- Is written out and practiced

Your turn!

Dr. Marina Sirota, Associate Professor at UCSF, joins you on an elevator at UCSF. You see an opportunity to impress her because you'd like a job in her lab someday.

You say: "Dr. Sirota, I'm in the AI4All group and really liked your lecture last week."

She says: "Thanks, I really enjoy working with AI4All. Tell me about ***YOU.***"

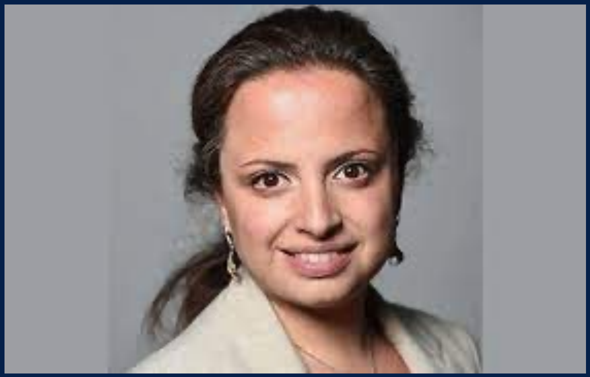

What do you say in 15-30 seconds before the elevator doors open and she walks away?

Breakout rooms in pairs. One minute each!

What is an Elevator Pitch? For our purposes:

- Any very brief statement about <u>you</u> and/or <u>your work</u> and/or <u>goals</u> and/or <u>needs</u>

- Content, length, style varies based on audience and purpose

- Designed to open further conversation…not to tell everything

- Follows a structure, outline or framework

- Is written out and practiced

Elevator pitch: "Tell me about yourself." (15-30 sec)

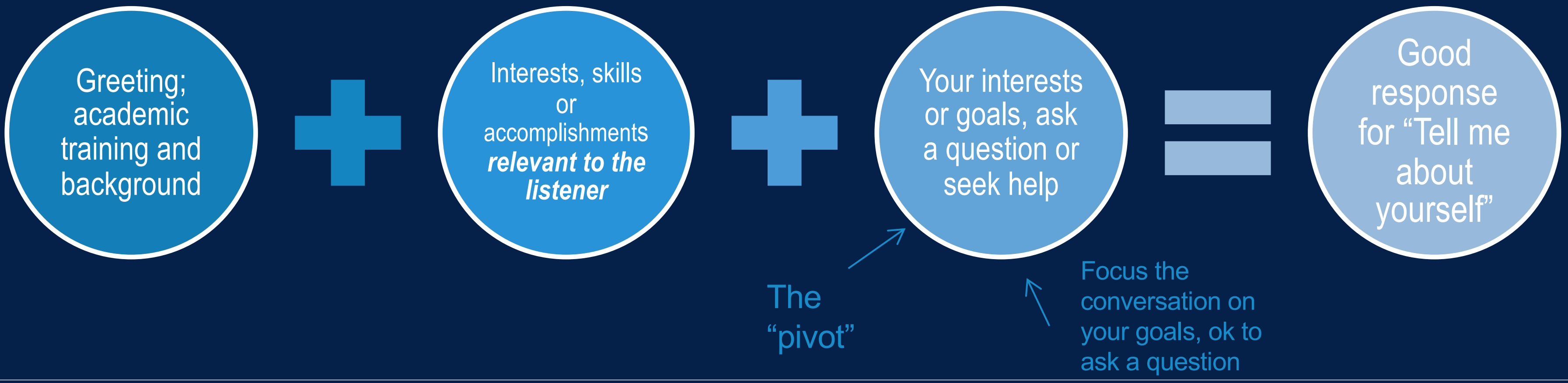

Elevator pitch: "Tell me about yourself." (15-30 sec)

My name is…I'll be a senior at Oakmont High, focusing on the science core

+

I joined the AI4All program because I wanted to see if I could apply my Python skills to a real human problem…

+

This coming year, I'd really like to be able to get some research experience. Do you have any advice for me?

=

Good response for "Tell me about yourself"

Greeting; academic training and background

+

Interests, skills or accomplishments ***relevant to the listener***

+

Your interests or goals, ask a question or seek help

=

Good response for "Tell me about yourself"

The "pivot"

Focus the conversation on your goals, ok to ask a question

Speed Practice and Critiques

In pairs: Decide who speaks first

Partner #1 delivers the elevator pitch (15-30 seconds)

Partner #2 offers critique (one minute or so)

- -Balance positive and constructive feedback
- -Clarity of description
- -Organization/flow
- -How could it be more engaging?

At the 2-minute bell, switch roles and repeat

Stop, refine, practice again

Applying the Elevator Pitch concept to College Interviews

- Often include questions about non-academic aspects of your background.
- Basic principle: Past behavior is the best predictor of future behavior.
- Questions are about your past behaviors, to see if you have demonstrated *behaviors considered critical for success in the future*
- **Behavioral questions:** designed to help the interviewer learn how you handled a situation in the past, so the interviewer can understand how you will respond in the future.

Applying the Elevator Pitch concept to College Interviews

What's the most effective way to respond to tough interview questions?

Tell a brief, structured example of a past success

Technique is called behavior-based story telling

Behavior-based interview questions usually start with:

- “Tell me about a time when…
- “Give me a past example of a situation when you…”

Behavior-based Interview Questions for College Admissions

Questions focused on your past behaviors

Past behavior is the best predictor of future behavior!

3 Behavior-based Interview Questions

- Give me an example of a time when you had to manage competing priorities effectively.
- Talk about a time when you had to be a leader.
- What is an area of weakness for you in school and how have you overcome it?

Responding to Behavior-based Interview Questions

Use the S-T-A-R framework to tell an organized, past-tense story

- *What is an area of weakness for you in school and how have you overcome it?*

Situation

Task

Action

Result

Responding to Behavior-based Interview Questions

Use the S-T-A-R framework to tell an organized, past-tense story

- What is an area of academic weakness for you and how have you overcome it?

Situation	• I struggle to write essays, and the quality of my writing as well as the time management was a problem in the past. I recognized this during the last year in my comparative geography class, when we had an essay due every Friday. I always meet my deadlines, but the first three weeks of the quarter I was up all Thursday night to get it done and my scores on those first three essays weren't great.
Task	• So I knew that I had to find a way to manage my time and also improve my writing skills or I was not going to get a good grade in the class.
Action	• So I took three actions to overcome this weakness. First, I talked with the teacher and she gave me a framework that social scientists use for making arguments and writing supportive paragraphs in social sciences papers. It was like a step-by-step formula for outlining my weekly essays. Second, I promised myself to complete a rough draft by Monday night each week. Finally, every Tuesday I went to the peer editing office during my free hour, to get feedback and editing help on my rough draft, and that left me two days to finish the essay and incorporate the edits.
Result	• After making those changes in my writing process, I pulled an A out of the class, and managed to have no more all-nighters.

Responding to Behavior-based Interview Questions

Practice the STAR format

- Select one behavioral-based question from list
- Write out your response in bullet-points, following the STAR format
  - *Talk about a time when you had to step up and be a leader.*
  - *What is an area of weakness for you in school and how have you overcome it?*

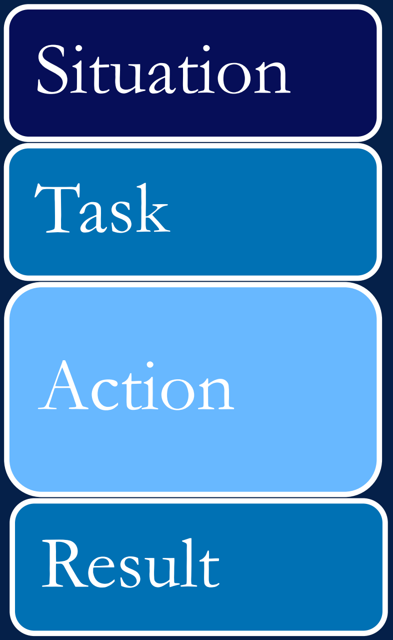

Interpersonal Skills and Team Fit

Practice the STAR technique

- Practice and critique with a partner
  - Feedback:
    - What did you like most about your partner's example?
    - How could your partner's story more clearly demonstrate the behavior?
    - How could your partner improve each section: Situation? Task? Action? Result?

Situation

Task

Action

Result

Applying the Elevator Pitch concept to your final project presentations

- Your group will have to give a presentation about your research project

- How will you introduce your group and your project in a way that captures audience attention and interest?

- Use an outline or framework to provide structure and keep yourselves brief and on track

Outline for a polished, professional introduction during your research project presentation	Your group’s introduction
Introductions of speaker(s) and topic or title	
Interesting fact, or problem description, or question, designed to capture interest	
What your group is working on, what you are trying to do, or even your hypothesis, what you hope to find	
Tell an overview of what you are going to tell the audience during the rest of your presentation.	

Outline for a polished, professional introduction during your research project presentation	Example
Introductions of speaker(s) and topic or title	Good morning. My name is Bill Lindstaedt, and this is Naledi Saul and Gabriela Monsalve. **We're going to talk about** some work we've been doing in the area of cholesterol management drugs.
Interesting fact, or problem description, or question, designed to capture interest	**Did you know** that nearly 10 percent of the US population – 30 million people - are taking a class of cholesterol-lowering drugs called statins? These drugs are effective but many patients report really severe muscle pain after starting to take statins.
What your group is working on, what you are trying to do, or even your hypothesis, what you hope to find	**In our group, we're working on** separating the cellular pathways leading to the beneficial effects of statins, from muscle toxicity mediated by statins. **We hope to** identify new drug molecule(s) that only activate the pathways that are healthy for the heart.
Tell an overview of what you are going to tell the audience during the rest of your presentation.	**In our presentation, we are going to talk about** how we've used various cell-based assays to identify a drug combination that mimics the good effects of statins but is devoid of the muscle toxicity associated with their use.
