# Supplementary material for: Nurturing diversity and inclusion in AI in Biomedicine through a virtual summer program for high school students
Source: PLoS Comput Biol. 2022 Jan 31;18(1):e1009719. doi: 10.1371/journal.pcbi.1009719 (PMC8830787; doi:10.1371/journal.pcbi.1009719)
Supplement: S5 Text — Descriptions of the 5 research projects for the 2020 program with links to code repositories and available data. (DOCX) [file pcbi.1009719.s009.docx]

**Supplementary Material 5 – Research Projects**

Abbreviations

AI: Artificial Intelligence

CNN Convolutional Neural Network

COVID-19: Coronavirus Disease 2019

HCV: Hepatitis C Virus

HEK293T: Human embryonic kidney 293 cells of variant expressing a temperature-sensitive allele of the SV40 T antigen

HIV: Human Immunodeficiency Virus

HPV: Human Papilloma Virus

PPI: Protein-Protein Interaction

LSTM: Long Short-Term Memory

MiST: Mass Spectrometry interaction STatistics

ML: Machine Learning

MWU: Mann Whitney U

PCA: Principal Component Analysis

PCR: Polymerase Chain Reaction

RNA: Ribonucleic Acid

SARS-CoV-2: Severe Acute Respiratory Syndrome Coronavirus 2

SVM: Support Vector Machine

TA: Teaching Assistant

TSNE: t-Distributed Stochastic Neighbor Embedding

UCSF: University of California, San Francisco

### **Project 1: AI for Global Health - AI and COVID-19 Time Series Diagnosis Data**

Students learned how to develop machine learning algorithms with utility for lower middle-income country (LMIC) settings. Their objective was to develop an algorithm that can predict the number of cases in a given country. Students used publicly available daily time series data describing confirmed COVID-19 infections and deaths per country and states across the world (over 266 regions) aggregated from the Johns Hopkins Center for System Sciences downloaded on July 1, 2020 (<https://github.com/CSSEGISandData>) [[1]](https://www.zotero.org/google-docs/?6QqKKb). Each student then manually pre-processed the dataset to a format in which they could conduct exploratory data analysis. The educational approach was to allow students to have first-hand experience in discovering the optimal way to plot and analyze various features of the data they were working with by experimenting with different visualization libraries and troubleshooting together real-time through video conferencing. Recognizing the diversity in time series trends between countries during exploratory data analysis, students chose to narrow the scope of the problem to focus on a specific country, selecting India due to its large number of cases and disparity in public health services. Students then did a literature review to understand the public health issues in India and how to design an algorithm that may actually provide utility to key stakeholders in the region.

Visualizing the trend of confirmed infections in India, they decided to develop a forecasting algorithm that can aid in identifying how many resources a given country or state will need. Students were then presented with high-level information on several ML techniques used for time series data analysis, such as autoregression [[2]](https://www.zotero.org/google-docs/?CR4cfD), Holt-Winters [[3]](https://www.zotero.org/google-docs/?MpPNrN) exponential smoothing, and neural networks [[4]](https://www.zotero.org/google-docs/?L8ZBrd). Following a group debrief, students were allowed to select modeling approaches that interested them. Afterwards, they trained, developed, and tested three different algorithms: autoregression, feed-forward neural network, and Long Short-Term Memory (LSTM) recurrent neural network (**S1A Fig, S1B Fig**).

Students first started with a simple ML technique for time series data, known as autoregression. Afterwards, they decided to see whether it was possible to leverage data from other countries that may be useful in the same prediction. They developed a feedforward neural network algorithm that leveraged data from 266 other countries/regions and predicted the most recent 5 days of COVID-19 cases in India. Students discovered that this model performed worse than the simple ML technique (**S1C Fig**). The students became interested in trying LSTM recurrent neural networks, due to their unique ability to model time-series data better than feed-forward networks. They trained the model to predict the next 5 days of data from the most recent 15 data points, and found it performed slightly better than the feed-forward, but not as well as the simple ML technique (**S1C Fig**).

Code can be found [here](https://colab.research.google.com/drive/1cBj53sMWilda8VhfUG07zSxc85Jcqlet?usp=sharing), https://drive.google.com/drive/folders/1_uKEeyzZ7NSmvG6JjkgocPbwyUToVp3J?usp=sharing


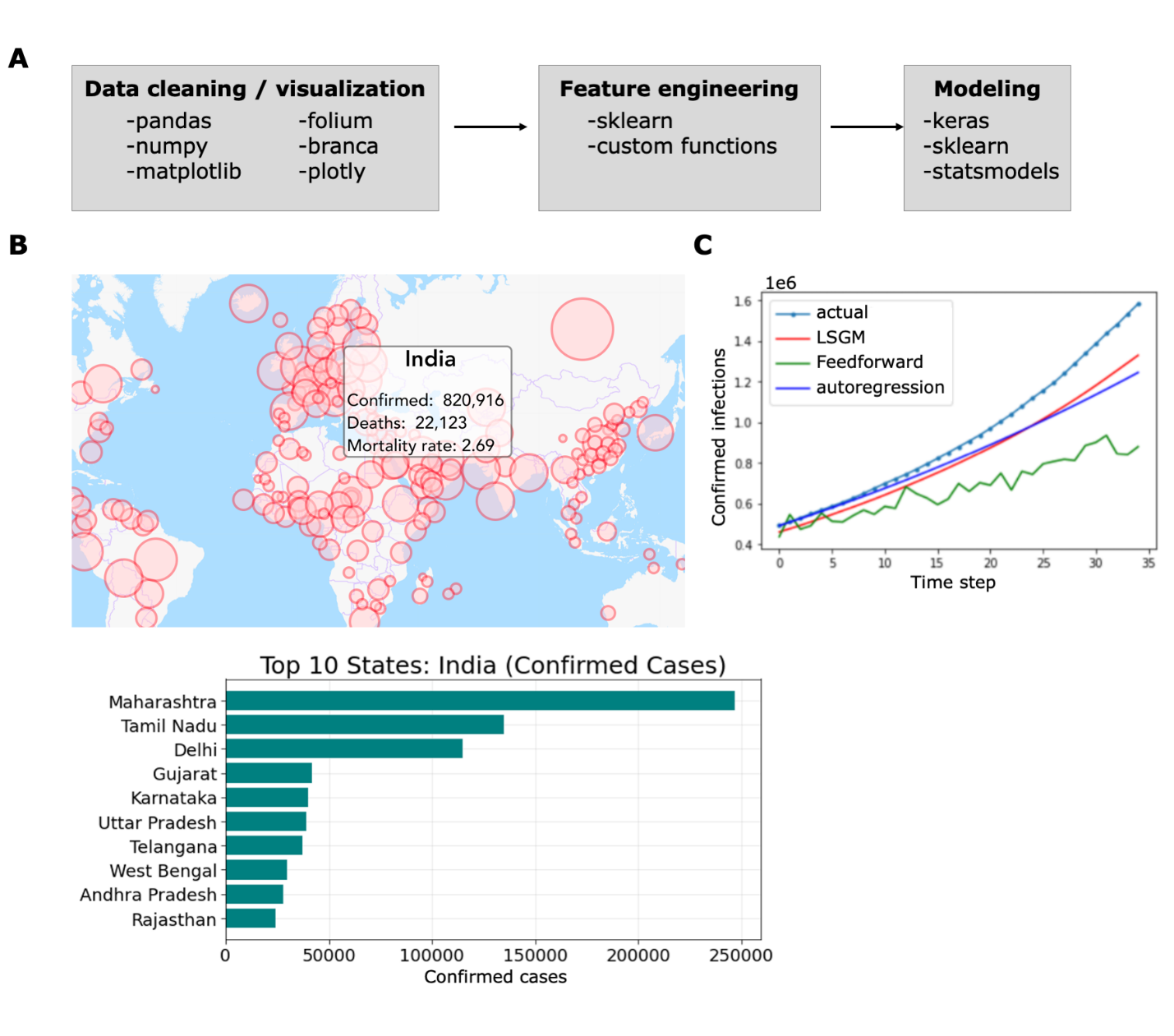


**S1 Fig. AI for Global Health. S1A Fig.** Schematic of major ML skills explored with packages/utilities used for instruction. **S1B Fig.** Examples of data visualizations created by students (base layer: <https://osm.org/go/wePI--?layers=T>, copyright OpenStreetMap contributors). **S1C Fig.** Model predictions compared to actual India COVID-19 data. Mean average percent error was 8.23%, 10.08%, 82.35% for autoregression, LSTM, and feedforward neural networks respectively.

### **Project 2: AI and Proteomics *-* COVID-19 Protein-Protein Interactions (PPI) predictions**

Students learned to implement supervised learning techniques to predict host protein interactors, given primary amino acid sequences of various viral proteins, and to test if proteins of similar sequences would interact with the same host proteins. The dataset was curated from two publicly available sources - 1) a host-pathogen protein-protein interaction (PPI) data in HEK293T cells for HIV [[5]](https://www.zotero.org/google-docs/?1JaFdL), HCV [[6]](https://www.zotero.org/google-docs/?pJJZLP), HPV [[7]](https://www.zotero.org/google-docs/?00Wf7A), Ebola [[8]](https://www.zotero.org/google-docs/?WwgRf0), Dengue [[9]](https://www.zotero.org/google-docs/?eRGQAV), and Zika [[9]](https://www.zotero.org/google-docs/?TNezcV), which contains sequence information on virus proteins with corresponding human protein information and their MiST scores, i.e. their interaction confidence scores [[10]](https://www.zotero.org/google-docs/?vPP0u7) and 2) human proteome FASTA files containing one protein sequence per gene [[10]](https://www.zotero.org/google-docs/?MceCCP). Mostly, project time was spent covering data processing, support vector machines (SVMs), and deep learning using Python. The six students chose one of six pathogens (HIV, HCV, HPV, Ebola, Dengue, and Zika) to work on individually. The students coded separately on personal Jupyter notebooks but shared code through CoLaboratory notebooks and collaborated through project time discussion, screen-sharing, and online messaging / chat messaging.

In the first week, the students accessed the PPI dataset of host-pathogen PPI data containing virus bait protein, corresponding human prey protein and gene name from PubMed for their chosen pathogen. In their first dataset, they mainly organized the bait and prey sequences and corresponding MiST score in a comprehensive and cohesive format. They collaboratively processed the primary PPI dataset by isolating their virus’ bait and protein sequence to build their virus-protein dataframe. To close off the week, the instructors introduced the second training dataset consisting of each of the six pathogens’ protein ID and sequences. The students downloaded and utilized FASTA files from UniProt that contained the protein ID and sequences for HIV, HCV, HPV, Ebola, Dengue, Zika and spent the remainder of project work time understanding the relationship between prey and protein sequences.

To start the second week, students learned about different sequence alignment algorithms. First, the students split each pair of interacting virus bait and human prey depending on the MiST score into positive (MiST >= 0.75) and negative (MiST < 0.75) datasets. After splitting the dataset, the instructors guided the students in constructing a data processing pipeline prior to building their predictive model. The group utilized a global pairwise alignment algorithm from Biopython [[11]](https://www.zotero.org/google-docs/?ysmiCF) to add the sequence alignment scores for each bait and prey pair to the positive and negative dataframes and decided on features that may serve as potent predictive variables including bait protein length, amino acid counts, and the atomic weight of the bait protein. Finally, the students visualized the distribution of alignment scores for the positive and negative data and evaluated the association.

In the final week, the group began creating and testing their machine learning models. The group implemented SVMs and collaboratively built their classifier. Students selected an 80% and 20% split for their training and testing data, respectively. Each student first trained their model using their individual virus data. Then, they trained the model using all their virus data to predict the interaction between each SARS-CoV-2 protein and each human protein from the first PPI dataframe they built. The students finetuned the algorithmic parameters, to improve the model’s performance. To visualize the algorithm’s optimal performance, each student built a confusion matrix for the SVM predicting virus-human protein interaction (**S2A-E Fig**) and extracted feature importance in a bar plot (**S2F Fig**). Additionally, students were guided by their instructors to build a convolutional neural network (CNN) for their individual pathogen.

Finally, the students spent the remainder of the last week investigating the implications of their research. The instructors presented ways that this type of data can be leveraged for drug discovery and repurposing whereupon the students discussed the implications of their experimental results on the COVID-19 pandemic.

Code availability, data, slides, and figures can be found [here](https://github.com/ucsfai4all/ucsfai4all2020_ppi_sars-cov2.git), https://github.com/ucsfai4all/ucsfai4all2020_ppi_sars-cov2.git

**
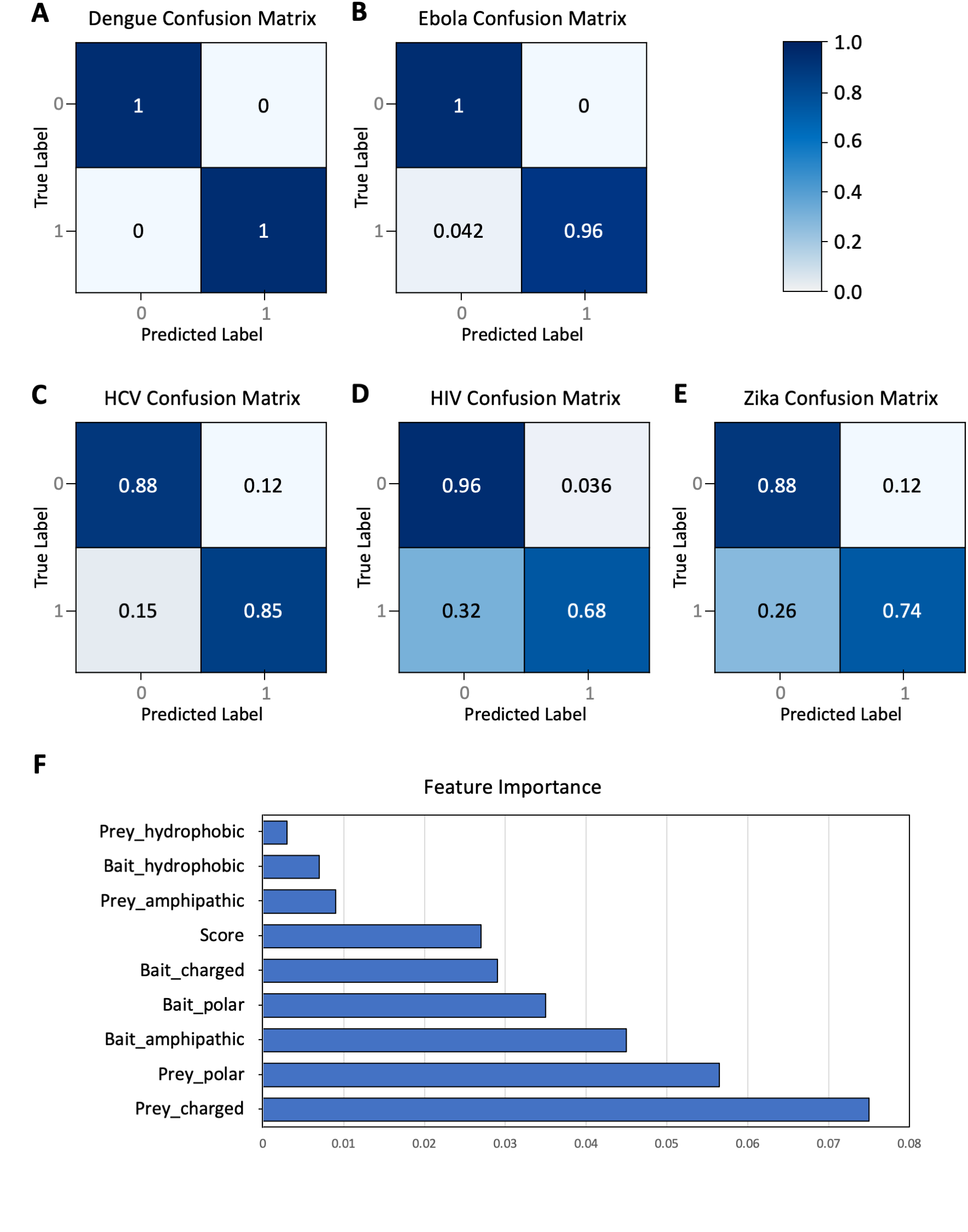
 S2 Fig. AI and Proteomics: COVID-19 Protein-Protein Interactions (PPI) Predictions. S2A-E: Confusion matrices visualizing the individual SVM models’ performances.** Evaluated the true and predicted virus-human protein interactions for Dengue, Ebola, HCV, HIV, and Zika viruses respectively. **S2F: Bar plot of feature importance extracted from Ebola virus trained SVM on SARS-CoV-2 virus data.** The prey proteins with a net positive or negative charge had the highest feature importance.

### **Project 3: AI for Imaging Data - Computer Vision for COVID-19 Chest X-Ray Classification**

Students learned to implement machine learning models that can classify COVID-19 cases in chest x-ray images. The dataset was curated from publicly available chest x-ray images datasets [[12,13]](https://www.zotero.org/google-docs/?7C5vMB) and contained 438 images from patients diagnosed with COVID-19 as well as 438 images from patients without pathologic findings, labeled as ‘no finding’ (**S3A-B Fig**). The instructors introduced concepts in data processing, computer vision, and deep learning using Python. The students applied these concepts in a hands-on project where they first visualized and evaluated the dataset then trained a convolutional neural network (CNN) to identify COVID-19 cases. The students coded separately on personal CoLab notebooks but collaborated and debugged together to the extent possible through online video discussions and screen-sharing.

The group began by examining the dataset through exploratory data analysis. They experimented with different data visualization approaches and packages to plot the distribution of attributes in the dataset. Using the visualizations, the students discovered potential biases in the dataset such as skewed gender distribution and different x-ray views for the COVID-19 images versus the ‘no finding’ images. The students also learned to use dataframe manipulations to survey subsets of the datasets. Together, they identified image features, such as lung region opacity and body outlines, that could be useful or potentially problematic for the classification task.

Next, the students developed fully connected and convolutional neural networks (CNN) using

PyTorch to perform binary classification. The instructors provided a starting code framework that guided the students in constructing a typical data processing and machine learning pipeline. The students completed the code by implementing missing core sections. They tracked model training speed as well as accuracy and loss curves to gain insight into the model training process (**S3C-D Fig**). The students also calculated metrics, such as F1 scores, and visualized latent space features to evaluate the model (**S3E Fig**). By discussing these analyses, the students identified areas where the model is performing poorly (e.g. incorrectly classifying COVID-19 images) and formulated hypotheses for potential reasons, such as the large variability in the COVID-19 images.

In the final week, the students branched out to work on follow-up ideas. For this half of the project, the students were largely self-driven to explore ideas or questions that interested them. The instructors helped the students work through code issues and brainstormed solutions. The follow-up ideas included iteratively improving CNN models by tuning hyperparameters and training other types of classifiers (SVM, regression) using features extracted by a pre-trained DenseNet model. One particular challenge the students tackled was model robustness. Using gradient class activation maps to provide interpretation of model decisions, they had observed that the CNN models were detecting regions outside of the lung (**S3F Fig**). This became a focus for model improvement. During breaks, the instructors also briefly introduced interesting papers and recent results in AI vision research. The group discussed challenges of interpretable decision-making and model brittleness, which complemented the tasks they were working on.

Code is available, https://github.com/ucsfai4all/ucsfai4all2020_computer_vision.git


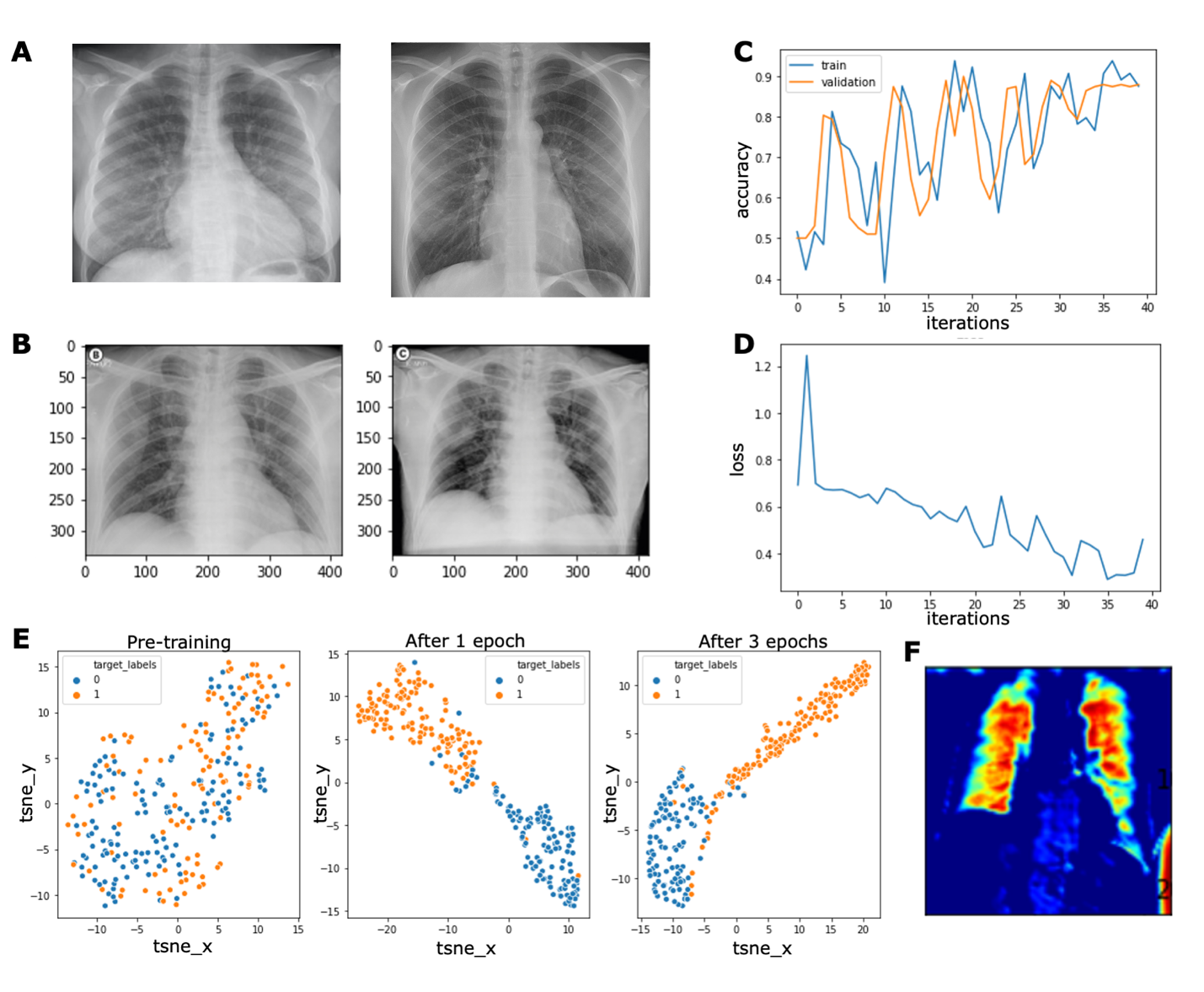


**S3 Fig. AI for Imaging Data. S3A. Examples of no finding images.** Credit: (left) Balasubramanya and Gulati (StatPearls, 2021)[15]. (right) Mikael Häggström (Wikipedia, 2017) [16]. **S3B. Subset of COVID-19 images.** Credit: Lim et al (J Korean Med Sci, 2020)[[14]](https://www.zotero.org/google-docs/?6yr9wS). **S3C. Accuracy graph during model training. S3D. Loss graph during model training. S3E. TSNE visualization of model training over time.** Blue represents no finding images while orange represents COVID-19 images. **S3F. Grad-CAM interpretation of CNN features.** Red in the heatmap represents greater importance while blue represents the least.

### **Project 4: Latent Variable Modeling of COVID-19 Metagenome Transcriptomic Profiles**

Previous analyses of metagenomic sequencing data from upper airway samples of 238 patients revealed a diminished innate immune response in patients positive for SARS-CoV2 through differential expression analysis, gene set enrichment analysis, and *in silico* estimation of cell type proportions [[17]](https://www.zotero.org/google-docs/?Z5TbcR). We hypothesized decomposition of metagenomic next generation RNA sequencing (mNGS) to reveal separable clusters of patient subgroups.

First, students accessed publicly available mNGS data and performed exploratory analysis on the study cohort of 94 patients who tested positive for SARS-CoV2 by gold standard clinical PCR. Evaluation of viral coinfection PCA embeddings led the students to hypothesize a correlation between binary coinfection status and SARS-CoV2 viral load. The students reasoned that patients with additional viral infection(s) may be generally more susceptible to SARS-CoV2 replication due to alteration of immune response dynamics under coinfection conditions. Patients were stratified into 2 groups: those with measurable alternative viral load, and those with sole viral metagenome alignment to SARS-CoV2. Welch’s t-test was applied to the average reads-per-million (rPM) of SARS-CoV2 between the two groups, and a significant difference below an error tolerance of 0.05 was found (p=1.947*10-4) (**S4A Fig**).

The students followed up this result with an analysis for confounding variables. In group discussion, we posited that samples with a large SARS-CoV2 viral load cause a higher frequency of alignment errors to evolutionarily similar genomes. To test this, the students inspected correlation coefficients between SARS-CoV2 and alternative, coinfection viral read abundance. The most correlated viruses were all coronaviruses, lending evidence to the hypothesis that large SARS-CoV2 viral load results in a higher proportion of reference alignment errors to genomically similar viruses (**S4B Fig**). This experience underscored the adage, “correlation does not imply causation,” lending a valuable lesson to the analysis of SARS-CoV2+ patient subgroups. Our analysis code is available (https://drive.google.com/drive/folders/1HXvWu-O_StXOdO5Mz63ZtW426bTUoxJ_?usp=sharing).


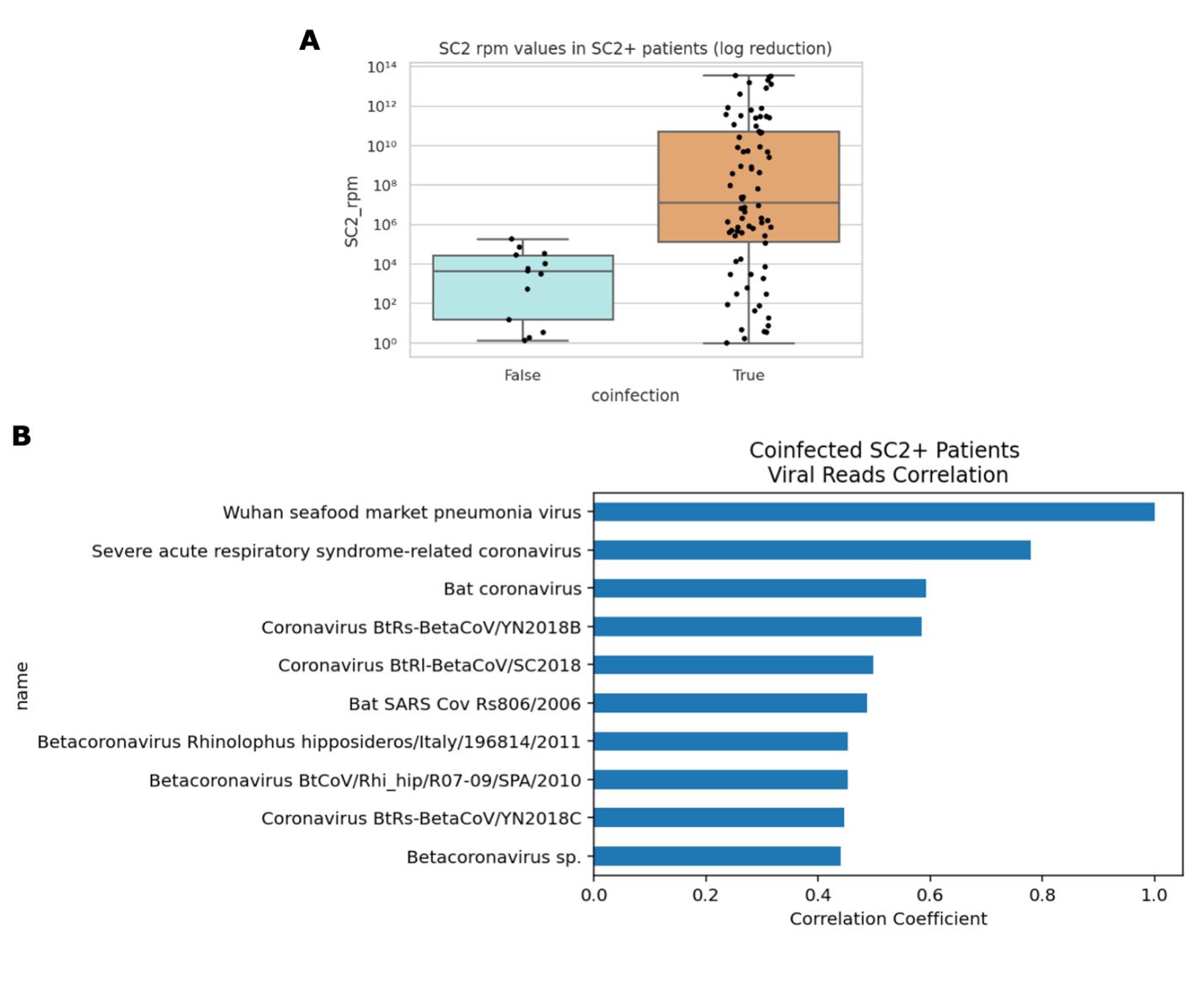


**S4 Fig. Latent Variable Modeling of COVID-19 Metagenome Transcriptomic Profiles.**

**S4A. Boxplot of SARS-CoV2 rPM by coinfection status.** Reads-per-million of SARS-CoV2 stratified by patients with alternative (orange) and no (blue) detectable viruses.

**S4B. Correlation coefficients between reads of SARS-CoV2 and coinfected viruses.** Highly correlated viruses are coronaviruses.

### **Project 5: PredictCOVID - AI and Electronic Medical Record (EMR) data**

Using real world data from (<https://www.kaggle.com/einsteindata4u/covid19>) [[18]](https://www.zotero.org/google-docs/?GFEgTb), students learned how to apply AI to (A) predict whether a patient is COVID positive or negative and (B) predict the severity of the COVID infection (i.e. admission into the general ward, semi-intensive care unit, or intensive care unit). The dataset included de-identified data from 5,644 patients as well as respective COVID-19 test results, patient age quantile, hospital admission ward, and various laboratory results from blood tests, urine tests, and pathogen tests from these patients. All data was normalized by standardizing to a mean of 0 and unit standard deviation. In the first week, students were introduced to the benefits and drawbacks of publicly available data, such as sources of bias and the need for intensive data preprocessing before the data can be utilized. Students took different approaches to data cleaning and imputation of missing values for input into machine learning models.

In the second week, students implemented and evaluated the performance of machine learning models on the varied input data, including but not limited to evaluating metrics of accuracy, area under the curve (AUC), and distribution of false negatives and positives (**S5A Fig**). Additionally, students were asked to go above and beyond to apply their findings to translational applications. For example, students were asked to critically evaluate the cost of false negatives (spreading COVID-19, not receiving treatment on time, worse outcomes) and false positives (waste of limited resources) in respect to patients and outcomes, and applying this evaluation to the decision of a model. Students were also asked to perform covariate analyses to determine feature importance and consider clinical relevance and application (**S5B-C Fig**). One finding that the group reported was that leukocytes were heavily negatively correlated with COVID test results (**S5D Fig**). Lastly, the group summarized their findings and recommendations for future plans to the entire group as well as the limitations and biases in the analyses and data (i.e. single location, limited follow-up, missing data).

Code is available, https://drive.google.com/drive/folders/1cZPvtSZY47dYA6Q1zhIhI9e7FRQj8kgR?usp=sharing

**
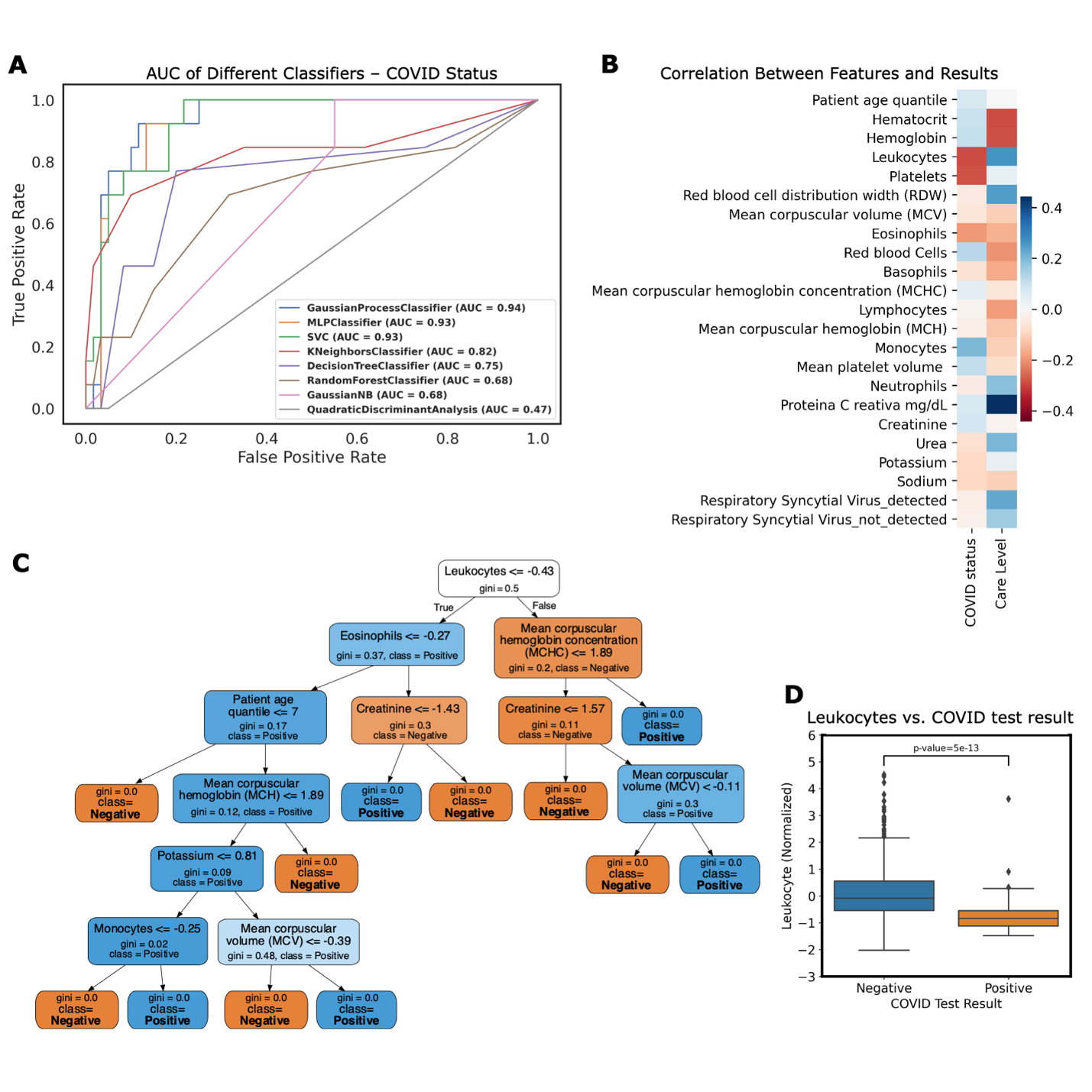
**

**S5 Fig. PredictCOVID: AI for deriving insights from healthcare data. S5A. Area Under the Curves (AUCs) of different machine learning classifiers for predicting COVID status from patient data.** AUC was one of the metrics used by the students to evaluate the performance of their machine learning models. **S5B. Decision tree classifier for COVID status.** Features used in decision making in each level of the tree are shown, with COVID status (positive/negative) at the leaves of the tree. **S5C. Heatmap of the correlation between clinical features and COVID status or Care Level.** Blue represents positive correlation while red represents negative correlation. **S5D. Boxplot of normalized leukocyte laboratory values and COVID status.** Leukocyte laboratory values negatively correlated with COVID test results.

# References

[1. Dong E, Du H, Gardner L. An interactive web-based dashboard to track COVID-19 in real time. Lancet Infect Dis. 2020 May 1;20(5):533–4.](https://www.zotero.org/google-docs/?Wudyqk)

[2. Akaike H. Fitting autoregressive models for prediction. Ann Inst Stat Math. 1969;21:243–7.](https://www.zotero.org/google-docs/?Wudyqk)

[3. Chatfield C. The Holt-Winters Forecasting Procedure. J R Stat Soc Ser C Appl Stat. 1978;27(3):264–79.](https://www.zotero.org/google-docs/?Wudyqk)

[4. Kröse B, Krose B, Smagt P van der, Smagt P. An introduction to Neural Networks. 1993.](https://www.zotero.org/google-docs/?Wudyqk)

[5. Jäger S, Cimermancic P, Gulbahce N, Johnson JR, McGovern KE, Clarke SC, et al. Global landscape of HIV-human protein complexes. Nature. 2011 Dec 21;481(7381):365–70.](https://www.zotero.org/google-docs/?Wudyqk)

[6. Ramage HR, Kumar GR, Verschueren E, Johnson JR, Von Dollen J, Johnson T, et al. A Combined Proteomics/Genomics Approach Links Hepatitis C Virus Infection with Nonsense-Mediated mRNA Decay. Mol Cell. 2015 Jan 22;57(2):329–40.](https://www.zotero.org/google-docs/?Wudyqk)

[7. Eckhardt M, Zhang W, Gross AM, Von Dollen J, Johnson JR, Franks-Skiba KE, et al. Multiple Routes to Oncogenesis Are Promoted by the Human Papillomavirus-Host Protein Network. Cancer Discov. 2018 Nov;8(11):1474–89.](https://www.zotero.org/google-docs/?Wudyqk)

[8. Batra J, Hultquist JF, Liu D, Shtanko O, Von Dollen J, Satkamp L, et al. Protein Interaction Mapping Identifies RBBP6 as a Negative Regulator of Ebola Virus Replication. Cell. 2018 Dec 13;175(7):1917-1930.e13.](https://www.zotero.org/google-docs/?Wudyqk)

[9. Shah PS, Link N, Jang GM, Sharp PP, Zhu T, Swaney DL, et al. Comparative Flavivirus-Host Protein Interaction Mapping Reveals Mechanisms of Dengue and Zika Virus Pathogenesis. Cell. 2018 Dec 13;175(7):1931-1945.e18.](https://www.zotero.org/google-docs/?Wudyqk)

[10. Breuza L, Poux S, Estreicher A, Famiglietti ML, Magrane M, Tognolli M, et al. The UniProtKB guide to the human proteome. Database J Biol Databases Curation [Internet]. 2016 Feb 19 [cited 2021 Feb 16];2016. Available from: https://www.ncbi.nlm.nih.gov/pmc/articles/PMC4761109/](https://www.zotero.org/google-docs/?Wudyqk)

[11. Cock PJA, Antao T, Chang JT, Chapman BA, Cox CJ, Dalke A, et al. Biopython: freely available Python tools for computational molecular biology and bioinformatics. Bioinformatics. 2009 Jun 1;25(11):1422–3.](https://www.zotero.org/google-docs/?Wudyqk)

[12. Cohen JP, Morrison P, Dao L. COVID-19 Image Data Collection. ArXiv200311597 Cs Eess Q-Bio [Internet]. 2020 Mar 25 [cited 2021 Feb 3]; Available from: http://arxiv.org/abs/2003.11597](https://www.zotero.org/google-docs/?Wudyqk)

[13. Irvin J, Rajpurkar P, Ko M, Yu Y, Ciurea-Ilcus S, Chute C, et al. CheXpert: A Large Chest Radiograph Dataset with Uncertainty Labels and Expert Comparison. Proc AAAI Conf Artif Intell. 2019 Jul 17;33(01):590–7.](https://www.zotero.org/google-docs/?Wudyqk)

[14. Lim J, Jeon S, Shin H-Y, Kim MJ, Seong YM, Lee WJ, et al. Case of the Index Patient Who Caused Tertiary Transmission of Coronavirus Disease 2019 in Korea: the Application of Lopinavir/Ritonavir for the Treatment of COVID-19 Pneumonia Monitored by Quantitative RT-PCR. J Korean Med Sci [Internet]. 2020 Jan 8 [cited 2021 Feb 25];35(6). Available from: https://doi.org/10.3346/jkms.2020.35.e79](https://www.zotero.org/google-docs/?Wudyqk)

15. Gulati A, Balasubramanya R. Figure, Normal chest radiograph in the PA and Lateral projections [Internet]. StatPearls Publishing; 2021 [cited 2022 Jan 12]. Available from: https://www.ncbi.nlm.nih.gov/books/NBK558976/figure/article-37405.image.f1/

16. Chest radiograph. In: Wikipedia [Internet]. 2021 [cited 2022 Jan 12]. Available from: https://en.wikipedia.org/w/index.php?title=Chest_radiograph&oldid=1049059075

[17.](https://www.zotero.org/google-docs/?Wudyqk)  Mick E, Kamm J, Pisco AO, Ratnasiri K, Babik JM, Castañeda G, et al. Upper airway gene expression reveals suppressed immune responses to SARS-CoV-2 compared with other respiratory viruses. Nature Communications. 2020 Nov 17;11(1):5854.

18. Diagnosis of COVID-19 and its clinical spectrum [Internet]. [cited 2021 Mar 9]. Available from: https://kaggle.com/einsteindata4u/covid19
